# Patient advocacy: barriers and facilitators

**DOI:** 10.1186/1472-6955-5-3

**Published:** 2006-03-01

**Authors:** Reza Negarandeh, Fatemeh Oskouie, Fazlollah Ahmadi, Mansoure Nikravesh, Ingalill Rahm Hallberg

**Affiliations:** 1 Faculty of Nursing and Midwifery, Zanjan University of Medical Sciences, Parvin Etesami St., Zanjan, Iran; 2 Faculty of Nursing and Midwifery, Iran University of Medical Sciences, Rasid Yasami st. Valiasr Ave. Tehran, Iran; 3 Faculty of Medical Sciences, Tarbiat Modarres University, Jalal Al e Ahmad st., Tehran, Iran; 4 Deputy Dean, Medical Faculty, Department of Nursing,157 SE 221 00, Sweden

## Abstract

**Background:**

During the two recent decades, advocacy has been a topic of much debate in the nursing profession. Although advocacy has embraced a crucial role for nurses, its extent is often limited in practice. While a variety of studies have been generated all over the world, barriers and facilitators in the patient advocacy have not been completely identified. This article presents the findings of a study exploring the barriers and facilitators influencing the role of advocacy among Iranian nurses.

**Method:**

This study was conducted by grounded theory method. Participants were 24 Iranian registered nurses working in a large university hospital in Tehran, Iran. Semi-structured interviews were used for data collection. All interviews were transcribed verbatim and simultaneously Constant comparative analysis was used according to the Strauss and Corbin method.

**Results:**

Through data analysis, several main themes emerged to describe the factors that hindered or facilitated patient advocacy. Nurses in this study identified powerlessness, lack of support, law, code of ethics and motivation, limited communication, physicians leading, risk of advocacy, royalty to peers, and insufficient time to interact with patients and families as barriers to advocacy. As for factors that facilitated nurses to act as a patient advocate, it was found that the nature of nurse-patient relationship, recognizing patients' needs, nurses' responsibility, physician as a colleague, and nurses' knowledge and skills could be influential in adopting the advocacy role.

**Conclusion:**

Participants believed that in this context taking an advocacy role is difficult for nurses due to the barriers mentioned. Therefore, they make decisions and act as a patient's advocate in any situation concerning patient needs and status of barriers and facilitators. In most cases, they can not act at an optimal level; instead they accept only what they can do, which we called 'limited advocacy' in this study. It is concluded that advocacy is contextually complex, and is a controversial and risky component of the nursing practice. Further research is needed to determine the possibility of a correlation between identified barriers/ facilitators and the use of advocacy.

## Background

The role of patient advocacy is not new for nurses. Historically, patient advocacy has been a moral obligation for nurses. During recent years, nursing literature has been focused on the advocacy role and nursing professions has adopted the term 'patient advocacy' to denote an ideal of the practice [1]. Nurses assume that they have an ethical obligation to advocate for their patients [2]. They also frequently describe their judgments and actions on behalf of a patient as "being a patient advocate" [3,4].

An examination of advocacy in the nursing literature reflects broad and at times different perspectives. Advocacy has been described in ethical and legal frameworks and, more recently, as a philosophical foundation for practice. It has also been described in terms of specific actions such as helping the patient to obtain needed healthcare, assuring quality of care, defending the patient's rights, and serving as a liaison between the patient and the health care system.

Although multiple factors influence the need for advocacy, it is generally true that someone in the healthcare environment must assume the role of client advocate, particularly for the client whose self advocacy is impaired. Generally, advocacy aims to promote or reinforce a change in one's life or environment, in program or service, and in policy or legislation. In healthcare delivery, these activities focus on health conditions, healthcare resources, and the needs of patients and the public [6].

Advocacy is usually employed by someone powerful on behalf of someone who has no power [7]. In situations of vulnerability, powerlessness, or being involved in difficult circumstances, the individual needs to be advocated. Failure to do so may put the person's rights, welfare or basic needs in danger. Mallik (1997) concludes from her review that the core condition which demands advocacy action is the vulnerability of the client in two respects: personal vulnerability from illness and also vulnerability to risks inherent in the institutional processes to which the client is exposed in the health care system [8].

When nurses advocate for patients, they face certain risks and obstacles associated with the settings within which they work [9]. Therefore, there is always the possibility that attempts to advocate for a patient can fail, and that nurses can experience many barriers when addressing the rights, choices, or welfare of their patients.

As Nahigian (2003) noted, despite the fact that a variety of studies have been conducted in many countries, such as Sweden (Segesten, 1993), Korea (Cho, 1997), Australia (Breeding & Turner, 2002), England (Ingram, 1998; Mallik, 1997, 1988; Mallik & Fafferty, 2000; Snowball, 1996) and the United States (Chronkhite, 1991; Cole-Schonlau, 1991; Fetsch, 1991; Hatfield, 1991; Sellin, 1991, 1995); and additional studies in recent years by Hellwig, Yam and DigGiulio, 2003; Kubsch et al., 2004), the factors facilitating and inhibiting patient advocacy have not been completely identified [10,5,11]. This points to the importance of conducting research to obtain nurses' viewpoints on the facilitators and barriers for patient advocacy. This article reports the findings about barriers and facilitators that Iranian registered nurses perceive affecting their advocacy role from a large-scale grounded theory study.

## Method

This research used a constant comparative method to analyze data collected through an extensive grounded theory study, enabling the researchers to discover, describe, and discuss the factors which influence nurses' patient advocacy role. The approach was selected for the study because patient advocacy takes place in a complex workplace relationship and social context [12]. Glaser (1998) believed that the grounded theory researcher sets out to discover patterns of behavior among particular groups of people in specific contexts. The key word is discovery; the research is exploratory allowing for identification of variables that will be integrated into a theory in a larger project where the purpose of research is to develop substantive theory. In a small-scale project, it is acceptable to describe and explain some underlying social processes shaping interaction and behavior [13].

### Context

Nursing care in Iran before 1915 was carried out by household women or servants. Hospitalized patients were also cared for by untrained personnel. Due to the lack of basic education, social and cultural status, and some religious limitations for women, nursing did not have much advancement during that time. Iranian qualified nursing began in 1916, when a three-year nursing school was established in Tabriz. After 1916 there was a gradual increase in nursing schools across the country. Before the Islamic revolution in 1979, the majority of nurses were female and cared for both men and women. After the revolution, the government decided that nursing schools allow entry of male students up to 50 percent of those admitted, according to the belief that men should care for men and that women provide care for women. In 1980, when the war between Iran and Iraq began this point of view has been continued for many years. But due to the fact that the nursing profession could be more suitable for females in Iranian culture, the number of male students in nursing schools has been declined gradually. At present, nurses (male and female) can study at the university level from a bachelor's degree up to a PhD degree.

### Sampling and data collection

The sample consisted of 24 nurses (staff nurses, head nurses and supervisors) working in a large university hospital in Tehran. Eighteen nurses, 3 head nurses and 3 supervisors were interviewed. The participants' age ranged from 23 to 50 with an average of 33.45. Nursing practice experiences ranged from 1 to 26 years with a mean of 10.59 years. Twenty three of participants had BS degree in nursing, and one participant had MS degree in physiology. Twenty one of participants were female and three were male. All the nurses who worked full-time in the period of study were considered as potential participants.

Purposeful sampling was used for the initial interviews and, according to the emerging codes and categories data was collected by means of theoretical sampling. We had planned to interview nurses with at least three years of work experience, however, emerging codes and categories, especially the codes related to desensitizing (working long time had made nurses grow desensitized), led us to interview a number of novice nurses. In total, 5 novice nurses were invited to be interviewed.

Interviews

The main researcher R. Negarandeh explained the aim of the study and the research questions for each potential participant. Upon accepting to participate in the research, and after signing the informed consent sheet, nurses were given an appointment for the interview. For the sake of participants' convenience, interviews were carried out at the time they felt their workload was lower and had enough time to be interviewed. Individual semi-structured interviews were conducted in a private room at the workplace. The interview guide consisted of core open-ended questions to allow the respondents to explain their own viewpoints and experiences as completely as possible. Each interview began with a broad question, such as "could you describe one of your working shifts?" Participants where then asked to explain their own experiences and perceptions of "patient advocacy", as well as "barriers and facilitators" that affected taking on the advocacy role. The interviews continued with the topic questions and probes in order to capture a deeper understanding of the phenomenon under study. All interviews were carried out by the same interviewer. Interviews were recorded by a digital sound recorder, transcribed verbatim and analyzed consecutively. The duration of interview sessions ranged from 40 to75 minutes, with an average of one hour, depending on participants' tolerance and their interest in explaining their own experiences.

### Data analysis

Data analysis resembles a discussion between the actual data, the created theory, the memos and the researcher. Such discussion takes place when the data are broken down, conceptualized and put back together in new ways. The data give rise to the codes and the categories which combine the codes. The categories and hypotheses must be verified against the data by comparing the categories with each other, with the data and with the researcher's conclusions [13].

Data from the interviews were analyzed concurrently using constant comparative method. Data analysis started at the same time with the data collection and each interview was transcribed verbatim and analyzed before the next interview took place. In other words, each interview provided the direction for the next one. The process of interviewing was stopped when data saturation occurred. Data were considered "saturated" when no more codes could be identified and the category was "coherent" or made sense.

Open, axial and selective coding was applied to the data [14]. Through open coding, the interview transcripts were reviewed several times and the data reduced to the codes and then the categories were formed from the codes, in a manner that similar codes were grouped into the same categories. The focus of axial coding was on specifying a category in the context in which it had appeared. This process allowed links to be made between categories and their subcategories, and then selective coding developed the main categories and their interrelations. If the researcher is simply concerned with exploring or describing the phenomena being studied, axial coding completes the analysis. Hence data analysis was stopped at this phase for the aim of this article. However, grounded theory, as the term suggests, seeks to go further. For this you need to go on selective coding.

Regarding trustworthiness, credibility was established through member check, peer check and prolonged engagement. The participants were contacted after the analysis and were given a full transcript of their respective coded interviews with a summary of the emergent themes to determine whether the codes and themes were suitable to their experiences. Then three expert supervisors and two other doctoral students of nursing conducted the peer checking. Prolonged engagement with the participants within the research field helped the main researcher to gain the participants' trust and a better understanding of the research fields. Maximum variation of sampling (in terms of the type of ward, years of working experience and place of duty) also enhanced the confirmability and credibility of data. This sampling strategy enabled the researcher to capture a vast range of views and experiences carefully [15].

The researchers made every effort to have a precise documentation of the direction of the research and the decisions made in order to save the "auditability" for the other researchers who would follow the direction of the research. Furthermore, results were checked with a number of nurses who had not participated in the study in order that they could confirm the fitness of the results.

### Ethical considerations

Ethical issues were concerned with the participant's autonomy, confidentiality and anonymity during the study period. All participants were informed of the purpose and design of the study and also the voluntary nature of their participation. The research proposal was approved by the Iran University of Medical Sciences Research Council. Informed consent was attained from the participants in writing and signed by them for all stages of the study. Moreover, an official permission was attained from the hospital director, nursing manager and head nurses in order to conduct the study.

## Results

Through the process of data analysis, several categories emerged that explain the process of patient advocacy and factors that act as barriers or facilitators to patient advocacy. The categories reflecting the barriers and the facilitators to patient advocacy are shown in Table 1.

**Table 1:** The Barriers and Facilitators to Patient Advocacy

**Barriers to patients advocacy**	**Facilitators to patients advocacy**
Powerlessness	Nurse-patient relationship
Lack of Law and Code of Ethics	Recognizing and paying attention to patients' needs and conditions
Lack of Support for nurses	Nurses' responsibility
Physicians leading	Physician as colleague
Time shortage	Nurses' knowledge and skills
Limited communication	
Risks of advocacy	
Loyalty to peer	
Lack of motivation	

### Barriers to advocacy

Participants cited *Powerlessness *as a key barrier to advocacy. The following examples illustrate this theme: "We are working as a team, but when a shortcoming or neglect happens at work, as a nurse with sufficient knowledge and practical experiences, I notice it, but we either do not talk about it properly, or would be too cautious whether to mention it or not".

Several nurses noted that *Lack of Law and Code of Ethics *act as barriers to advocacy role. Comments that reflected this include: "If there are some rules, we are still unaware of them or we are not mentioned".

*Lack of Support for nurses *was identified as another advocacy barrier. Participants felt that they did not receive any support for advocacy action from managers. Supervisors confirmed the nurses' statements as well. Some examples included the following : "To be an effective advocator, we need to be supported" or similarly another participant claimed that "No one supports us, for instance the head nurse or matron" or another nurse said: "We are not supported well, as a result, the patients can not be supported as well".

Almost all of the nurses believed that "*Physicians leading*" was the most important factor that produced obstacles to advocacy. For example one participant believed that: "It is very hard to talk on behalf of the patient, even having good knowledge of the matter. I'm not allowed to say, for example, oh, doctor you made mistake about that patient, in these cases, I don't know what will happen to me". Another nurse stated that: "In my opinion, the nurse has the largest part in patient advocacy, but this role is not considered here, because as I said there is a physician-leading system here; so, if we want to do more advocacy, it should be done in a concealed manner".

Informants also noted that *time constraints *forced them to revise work patterns to complete many tasks in a limited time. Examples include the following: "When you have a trolley full of medicine and you are still in room 1, perhaps a patient wants to have a conversation but time is pressing and there is still a long way to go before the job is finished, we cannot spare time for the patients even if we want to, time is very important".

*Limited communication *was also viewed as an important barrier for nurses to be as patient advocate. For example: "I have to say with the situation at the intensive care unit (ICU) and the patients we have, we don't have much time to sit down and listen to our patients, however listening to their expressions, talking about their conditions, family and disease courses can promote patient's spiritual status and reduce patient's stress". Or, "Now, the close relationship with patients has been replaced with recording processes. At first, nurses must have relationship with the patient and seek his/her needs to achieve patient's affairs" or "More time must be allocated to listen to the patient's words in detail. But instead, we just watch them and do certain routine treatments for them and at the end we write our report and that is it".

All of the nurses assumed that being patient advocate had unavoidable risks for advocators. Thus "*Risk of advocacy*" became a key determinant for accepting or refusing advocacy role. The following explanations illustrate this theme: "Who supports nurses' legally? You, as a teacher, would ask my nurse to become an advocator for the patient. If the nurse does so and then the hospital president sacks him/her, who will support this nurse?" or another participant believed that, "It is just impossible, you have absolutely no right to complain, and if you do so, your 30 hours overtime payment would be reduced to ten hours to stop your complaining".

Nurses that participated in this study cited "*Loyalty to peer*" as a barrier to patient advocacy too. The subsequent examples explain this feeling: "Listen to me, when we work together as a group, we cannot spoil each other in the system".

Finally "*Lack of motivation*" was also described as a critical barrier. The following example indicates this barrier: "So, all personnel are working with frustration and reluctance ... it is inevitable, the important point is that the management method and staffing strategies are the strongest determinants to quality care and advocacy".

### Advocacy facilitators

Informants also spoke about the factors that facilitated the practice of patient advocacy. The development of functional *nurse-patient relationship *was identified as a key factor to facilitating advocacy. Nurse-patient relationship recurred more than other themes in this study. From the participants' perspectives, establishing an appropriate relationship between a nurse and patients was necessary to patient advocacy. The quality of this relationship was described in the following examples: "I try to have a good relationship with them, listen to them carefully, and do as they wish" or "Most of our patients have one or more family members to accompany them. For instance, an old man who has had an eye surgery may also be cared by his daughter or son, but I strongly feel that my relationship with the patient is more important than family relationship for him and gives a more sense of security to him". Participants 11 can explain this better "... but the nurse-patient relationship is really close, very often nurses have closer relationship with their patients than their children ...".

"*Recognizing and paying attention to patients' needs and conditions*" was another factor that could facilitate patient advocacy. All nurses believed that comprehensive patient assessment enabled them to understand patients' real needs and be more effective in patient advocacy. They also believed that patients had different and varying needs and conditions; therefore, it was necessary for nurses to become aware of patient's needs and conditions in order to act on behalf of the patient. In this respect one participant said: "In spite of the fact that the social worker may refrain from supporting patients on the grounds that the patient has a family and enough resources, the patient needs a social worker. We are in the best position to persuade a social worker role that, for example, this patient has a broken up family and so on ... I mean we are frequently encountered with the issues the patient and his/her family may have. So, you, as a nurse, must assess the patient's situation and refer him/her to relevant social support resources".

Another theme that emerged from the data collected was "*Nurses' responsibility*" which could facilitate the patient advocacy. In the participants' narratives, nurses' responsibility and accountability were two factors that had an effect on advocacy role. Also, they believed that nurse's conscience, commitment to professional code of ethics, and respect of patient rights could facilitate patient advocacy. The subsequent examples explain this: "When my patient needs some medications and she/he doesn't have it, I call other wards frequently. For example, I had a child patient from Afghanistan and I paid attention to him very much, because he was a very little guy. When his antibiotic finished, I looked for medication in all wards of the hospital to provide it" and "I think that the origin of advocacy is mainly in conscience, I feel it stems from nurses' conscience and as well; it is strongly interconnected with this profession".

"Physician as a colleague" this mean that taking team approach to coordinating patient care and services was reported as a crucial factor, as the following examples illustrate: "But some doctors if you tell them what they are doing is wrong, they don't like it, in other words, some worry to tell them and in the case they feel they get offended and act harshly, but some physicians easily accept our comments". Many nurses noted the importance of developing a friendly relationship with physicians as a helpful strategy. "Over the years I have developed a respectful relationship with all the physicians and they accept what I say". Another nurse believed "This mutual collaboration between nurse and physician usually culminates in patient advocacy".

All nurses described the "*Knowledge and skills" *are essential to advocacy. Clinical knowledge and some skills were reported as crucial factors to effective advocacy. Participants in this study also believed that in-service education can improve their knowledge and skill that is needed to patient advocacy. One of participants said "In order to the nurse to be better advocate, he/she must improve his/her knowledge, and advance braveness and self esteem ...".

## Discussion

All nurses in this study believed that patient advocacy was one of the primary roles of the nurse. In the nursing literature advocacy is embraced as an essential component of practice, is based on nursing theory, is systematically implemented, and is influenced by several factors [11]. The data arising from this study provide evidence to support the barriers and facilitators to the advocacy process. In the study, many barriers and facilitators to patient advocacy from Iranian registered nurses' perspectives were determined. The factors that were identified as barriers to advocacy by the nurses were powerlessness, lack of support, lack of national law and code of ethics, limited communication, physicians leading, risks of advocacy, royalty to peers, lack of motivation and insufficient time to interact with patients and families. These findings were congruent with literature that describes the barriers to effective advocacy. Kohnke (1980) believes that the greatest obstacle to advocacy is the healthcare institution itself, because client advocacy is basically in conflict with the culture of the hospital system [16].

Hellwig, Yam, and DiGiulio (2003), in a phenomenological study found time constraints and doing more with fewer acts as main advocacy barriers [5]. Mallik (1977) noted it could be seen difficulties surround implementation of advocacy. More significantly, the concept and the role of patient advocate are open to a variety of different interpretations and, in practice, the power of outside professional groups, especially those of doctors and managers, makes it difficult for the individual nurse to operationalize the concept [8].

Problems related to job security and management conflicts were cited as barriers to nursing advocacy by Sellin (1995). She reported that much of an advocate's strength in acting comes from the support that is felt from coworkers, peers and nursing administrators. The director of nursing's attitude towards patient advocacy is a factor that influences unit culture about advocacy. The directors' attitudes toward advocacy, and the support that they show to assistant administrators and nurse managers in response to advocacy, help to decrease the degree of risk related to patient advocacy by staff nurses [3].

Rushton (1995) noted that in institutions where hierarchal decision making, objectivity, efficiency, and traditional power structures are valued, there may be incongruence between the stated philosophy, values, and goals of the organization and the reality of the work place. In such environments, conflicts related to nurse-physician relationships, institutional policies and practices, (particularly those that govern resource allocation and the quality of patient care), professional behaviors of administrators and colleagues, and job security arises. Advocacy can also be limited by real or perceived legal constraints, societal values and factors, the organizational culture of the institution where one works, nursing's status within the health care system, and political and economic constraints [16].

Davis et al. (2004) noted in their study, all except one nurse said that nursing leadership had a responsibility to help nursing staff to advocate for patients and families. All of these nurses noted that physicians had strong authority and they therefore realized their vulnerable position in the organizational structure of health facilities. They believed that without nursing leadership support for advocacy, patient care and protection could become secondary to self-protection [18]. Thus managers must promote morally sound judgment and behavior among staff. Advocacy for the best interest of the patient must be mentored among new practitioners so that moral behavior is a cultural expectation on a unit and within an organization [6].

Regarding factors that facilitate the nurses to act as a patient advocate, we found that the nature of nurse-patient relationship, recognizing and paying attention to patients' needs and conditions, nurses' responsibility, physician as colleague, and nurses' knowledge and skills can affect on adopting a patient advocacy role.

Several factors that could influence the use of advocacy were identified in the literature. Hellwig, Yam, and DiGiulio (2003), in a phenomenological study reported that through nurses' perceptions the main facilitator factor are physician support, utilizing a team approach, and rapport with insurance companies and other agencies [5]. As we interviewed the nurses, data revealed that their ability to be patient advocator varied at different situations and settings. Nurses, who could manage internal and external resources, might act better in patient advocacy role. In a qualitative study, Sellin (1995) found that the quality of nurse-patient relationship (quality and the length of relationship) can influence the use of advocacy role. Sellin also reported that nurses' personal and professional qualities could influence using patient advocacy [3].

"*Nurses' responsibility*" was another factor which could facilitate the patient advocacy. O'Connor and Kelly (2005) point out that Professional responsibility was a key trigger for advocacy. They also revealed the importance of nurses' strong relationship with patients, which provided a mandate to act in their best interests [17].

Rushton (1995) noted that effective leadership, open communication patterns, collaborative problem solving methods, compatibility of values and philosophy among various health care team members and procedural safeguards such as ethics committees facilitate advocacy and patient outcomes [16].

All respondents in this study believed that Iranian nurses need more knowledge to be able to undertake the advocacy role. O'Connor and Kelly (2005) wrote that the ability to advocate was also based on sound nursing knowledge and expertise [17]. Similar to this study's finding, Mattiasson and Anderson (1995) found a positive correlation between education and advocacy [19]. Ingram (1998) reported that the nurses, who attend ethics courses, would engage more advocacy situations, had a greater influence, and more effectively resolved ethical conflicts [20].

Davis et al (2004) found similar results amongst Japanese nurses. In their study, nurses believed factors such as patient centered care philosophy; physician as colleague; general democratic environment on ward; cooperative spirit among nursing staff members; and head nurse supports staff would promote advocacy [18].

Although the data provided a rich description for advocacy facilitators and barriers from participants' viewpoint, generalization of research findings to the larger population of nurses is limited. Results were also checked with several of the expert nurses who did not participate in the research and they confirmed the fitness of the results. Comments provided by these nurses also support the transferability of the findings presented in this study.

## Conclusion

This study illustrates the barriers and facilitators to patient advocacy from the Iranian registered nurses' perspectives. Participants in this study believed that in these circumstances and by taking into consideration the barriers mentioned, taking an advocacy role is difficult for nurses. Therefore, they make decisions and act as a patient advocate in any situation concerning patient needs and the status of barriers and facilitators. In most cases, they cannot act at an optimal level, instead they accept only what they can do, what we call this as 'limited advocacy'. Witts (1986, 1992) and Courtney (1985), in their study on ethical decision-making found that nurses in the United Kingdom did act as advocates, but that it happened informally and was taken up to the extent that circumstances allowed [8]. Chambliss (1996) who spent several years observing nurses who practiced in hospital settings has noted, "The nurse often knows what is the right thing to do, but is prevented from accomplishing this by institutional obstacles". Such obstacles can rarely be overcome by the efforts of single individuals [1].

It can be concluded that advocacy is contextually complex, and is a controversial and risky component of any nursing practice. Different workplaces and cultures may affect the findings of the study. This inquiry is a description of the barriers and facilitators of advocacy from the Iranian nurses' perspective, nurses working in others areas may have different views, or may experience similar barriers and facilitators to patient advocacy. Therefore, additional research studies are needed to further our understanding of the barriers and facilitators of patient advocacy in nursing. It is recommended that future quantitative research be conducted to identify the correlation between the identified barriers and facilitators and the use of advocacy, if any. In addition specific knowledge and behaviors that support the advocacy role should be examined.

## Competing interests

The author(s) declare that they have no competing interests.

## Authors' contributions

RN is main researcher that Initiated, designed, collected and analyzed the data and wrote the paper. FO, FA, MN and IRH were the co-researcher who helped in design and data analysis.

## Pre-publication history

The pre-publication history for this paper can be accessed here:


